# Treatment Outcome, Duration, and Costs: A Comparison of Performance Indicators Using Data from Eight Mental Health Care Providers in The Netherlands

**DOI:** 10.1007/s10488-017-0818-x

**Published:** 2017-07-22

**Authors:** E. de Beurs, E. H. Warmerdam, S. C. C. Oudejans, M. Spits, P. Dingemanse, S. D. D. de Graaf, I. W. de Groot, H. Houben, W. G. E. Kuyck, E. O. Noorthoorn, M. A. Nugter, S. C. C. Robbers, G. E. van Son

**Affiliations:** 1 SBG, Bilthoven, The Netherlands; 2 Leiden University, Wassenaarseweg 52, 2333 AK Leiden, The Netherlands; 3 Mark Bench/AMC-Psychiatrie, Amsterdam, The Netherlands; 4 GGZ-Altrecht Mental Health, Utrecht, The Netherlands; 5 VGZ, Eindhoven, The Netherlands; 6 Dimence Groep, Deventer, The Netherlands; 7 Mentaal Beter, Hilversum, The Netherlands; 8 GGZ-Parnassia-PsyQ, The Hague, The Netherlands; 9 GGNet, Warnsveld, The Netherlands; 10 GGZ-NHN, Heerhugowaard, The Netherlands; 11 Yulius Academy, Yulius Mental Health, Barendrecht, The Netherlands; 12 Department of Care en Quality, GGZ Rivierduinen, Leiden, The Netherlands

**Keywords:** Performance indicator, Benchmarking, Treatment outcome, Costs, Duration

## Abstract

Assessing performance of mental health services (MHS) providers merely by their outcomes is insufficient. Process factors, such as treatment cost or duration, should also be considered in a meaningful and thorough analysis of quality of care. The present study aims to examine various performance indicators based on treatment outcome and two process factors: duration and cost of treatment. Data of patients with depression or anxiety from eight Dutch MHS providers were used. Treatment outcome was operationalized as case mix corrected pre-to-posttreatment change scores and as reliable change (improved) and clinical significant change (recovered). Duration and cost were corrected for case mix differences as well. Three performance indicators were calculated and compared: outcome as such, duration per outcome, and cost per outcome. The results showed that performance indicators, which also take process variability into account, reveal larger differences between MHS providers than mere outcome. We recommend to use the three performance indicators in a complementary way. Average pre-to-posttreatment change allows for a simple and straightforward ranking of MHS providers. Duration per outcome informs patients on how MHS providers compare in how quickly symptomatic relief is achieved. Cost per outcome informs MHS providers on how they compare regarding the efficiency of their care. The substantial variation among MHS providers in outcome, treatment duration and cost calls for further exploration of its causes, dissemination of best practices, and continuous quality improvement.

## Introduction

The need to systematically gather data regarding process and outcome in health care to improve quality was first pointed out by Ellwood (1988), who suggested the creation of databases with treatment and outcome data by assessing patients at regular intervals. Ellwood’s suggestion was put into practice in a nationwide benchmarking initiative in mental health services (MHS) in The Netherlands, which took off in 2010. Treatment outcome data are collected through routine outcome monitoring (ROM), involving periodic assessments of outcome with self-report measures or rating scales (de Beurs et al. 2011). Thus, outcome scores during and after treatment have become available on a large scale.

Benchmarking in the Netherlands is facilitated by the Dutch Benchmark Foundation (Stichting Benchmark GGZ or SBG; http://www.sbggz.nl) (de Beurs et al. 2017). Aggregation of data collected through ROM allows for comparisons regarding the outcome of treatments between MHS providers (external benchmarking) and between teams or even individual therapists within MHS providers (internal benchmarking). Aggregated outcome information is useful for MHS providers and professionals working in MHS, but can also empower patients to make informed decisions regarding their choice of MHS provider (Hibbard 2003). Collecting data on outcome of treatment and combining this with information regarding the clinical or organizational process of therapy and information regarding patient characteristics, will allow us to learn what works best for whom (Paul 1967). Furthermore, these data can be used for quality improvement according to the Plan-Do-Study-Act approach (Deming 1950, 2000) of quality management (see also Taylor et al. 2014; Verbraak et al. 2015).

Evaluating treatment providers merely in terms of their outcomes is insufficient: MHS providers with similar outcomes may still differ in time and/or efforts required to attain these outcomes. To get a more complete picture of the state of affairs and to ensure a richer and fairer comparison between treatment providers, process aspects of the treatment, such as treatment length or costs should also be taken into consideration. Otherwise, practice variations in swiftness or efficiency may get obscured, whereas these aspects are highly relevant for the users of MHS—our patients—as well as for individual providers of care. The cornerstone of “value-based health care delivery” is increased value, that is outcome (patient based health outcomes) per money spent (Porter 2009; Porter and Teisberg 2006). Value (or quality) in health care is the best result (a beneficial outcome) for the best price. MHS providers strive for the best quality and to do so they need to be informed on how they compare to others regarding their effectiveness per money spent. Patients should also be enabled to seek the treatment of the highest quality. Another aspect of treatment quality, especially relevant for patients, is how quickly positive outcomes are achieved. Thus, for patients the duration of treatment is important. With equal outcome, a MHS provider is preferred who realizes improvement sooner rather than later.

Other factors, such as demographic or clinical features of the patients (e.g., gender, age, pretreatment severity, diagnostic complexity) may also influence the outcome, duration and cost of treatment. These factors are in general beyond control of the MHS provider. For a fair comparison, performance indicators need to be statistically corrected for these potential confounders (Iezzoni 2013). Therefore, case mix correction is applied to outcome, duration and cost.

The present study aims to investigate various performance indicators regarding their concordance and ability to discriminate between MHS providers. The main question is to what extent duration per outcome or cost per outcome discriminates differently between MHS providers than outcome per se. MHS providers may differ in outcome as well as in duration and cost of treatment. We hypothesize that these indicators converge: Length and cost are expected to be positively associated. They offer different perspectives on efficiency of care delivery relevant for different stakeholders. Duration per outcome—or how long it takes to get better—is relevant for the patient. Cost per outcome—or how much improvement is achieved per dollar spent—is mostly relevant for MHS providers and financiers, but for patients as tax and premium payers as well. Furthermore, duration and cost may also be associated with outcome: MHS providers with better outcomes may treat shorter and cheaper, as previous research has shown for individual therapists (Okiishi et al. 2003). If so, combining indicators into duration per outcome and cost per outcome will yield larger differences between MHS providers, as compared to outcome without taking duration or cost into account. Variation in efficiency of care may be revealed more easily by taking these numerators into account. Thus, we hypothesize a more precise discrimination among MHS providers with an index for efficiency rather than using outcome alone.

## Methods

### Outline of the Study

Anonymized data were obtained in an observational study in which performance of MHS providers was compared. SBG manages a large nationwide dataset covering outcome of full treatment trajectories of patients treated by MHS providers. Of these MHS providers, eight participated in a pilot project to study outcome of treatment corrected for process indicators. The MHS providers were ranked on outcome: the mean of case mix corrected pre-to-posttreatment change scores on symptomatology (attained after a full treatment trajectory). The MHS providers were coded henceforth 1–8 (with 1 having the best results). For the present study, treatment trajectories that started in 2013 or 2014, and concluded in 2014 were selected. Only treatment trajectories with complete pre- and posttreatment data were included (49% of all remunerated treatments, see Table 1). The maximum treatment length was 2 years, and covered 90% of all monitored treatments. For technical reasons, longer treatments (the remaining 10%) were excluded from the present study.

**Table 1:** Characteristics of the patients from the MHS providers

Provider	N	ROM^a^	Female	DEP	ANX	Age		SES		URB		GAF		Pretreatment T^b^	
		%	%	%	%	M	SD	M	SD	M	SD	M	SD	M	SD
1	104	77.6	72.1	52.9	47.1	37.0	11.7	3.43	1.45	2.77	1.13	58.1	6.8	54.9	9.1
2	1412	56.7	69.1	51.3	48.7	38.0	13.8	3.17	1.46	2.36	1.17	54.6	6.8	52.5	9.5
3	462	50.8	60.8	61.5	38.5	39.1	12.8	3.15	1.06	3.05	1.18	49.8	6.1	54.4	9.2
4	406	43.2	61.3	51.0	49.0	39.5	12.6	2.69	1.34	3.17	1.16	51.7	6.2	50.6	9.1
5	265	28.3	63.8	61.9	38.1	45.5	16.1	2.67	1.36	2.33	1.07	57.7	12.4	51.8	8.4
6	253	31.9	59.7	54.5	45.5	41.5	13.1	2.78	1.05	3.35	1.03	56.0	9.3	51.1	10.4
7	178	26.4	74.2	49.4	50.6	33.9	12.4	NA	NA	3.33	1.63	54.7	7.4	51.3	8.7
8	511	51.3	59.7	53.4	46.6	42.0	15.3	3.69	1.12	2.34	0.98	61.4	10.4	48.4	9.0
Total	3591	49.1	65.1	53.8	46.2	39.4	14.0	3.13	1.35	2.62	1.19	55.0	8.6	51.8	9.4

*NA* not available due to missing data

^a^ROM = ROM-response (percentage of treatments with complete pre-and posttreatment data) according to the benchmark reporting module (BRaM), website of SBG

^b^Bonferroni corrected pairwise comparisons: 1 > 2, 4, 6; 3 > 2, 5, 6, 7; 4 < 2, 3; 8 < 1 to 7

### Participants and Patients

The eight providers who participated in the study are among the 16 largest Dutch MHC institutes in terms of their yearly overall turnover and represent the various types of MHC providers in The Netherlands well: Six are large institutes where many clinicians—predominantly psychologists and psychiatrists—provide inpatient and outpatient care to all types of psychiatric disorders; two specialize in outpatient care of predominantly mood, anxiety, and personality disorders. The latter two providers are both franchise organizations operating nationally; one of these provided nation-wide data, the other provided data from a single municipality. There is quite some variation among providers regarding structural factors (some providers are large institutes providing a mix of inpatient and outpatient care, whereas other provide only outpatient treatment and claim to abide better to clinical guidelines for short problem-focused treatments) There is variation in process factors as well (possibly due to different theoretical approaches). Thus, we may expect variance in outcome and efficiency as well.

In order to homogenize the study sample and improve comparability of results, we selected patients with DSM-IV (American Psychiatric Association 1994) depressive disorders and/or anxiety disorders. The patients received predominantly outpatient psychotherapy and/or pharmacotherapy. Table 1 presents an overview of characteristics of included patients.

### Outcome Measures

Treatment outcome was assessed through the repeated use of various reliable and valid self-report questionnaires for general psychopathology. Five questionnaires were used: the symptomatic distress scale of the outcome questionnaire (OQ-45; Lambert et al. 2004); the total score of the depression anxiety stress scales (DASS-21; Lovibond and Lovibond 1993); the total score of the brief symptom inventory (BSI; Derogatis 1975); the total score of the short symptom list (Korte Klachtenlijst—KKL; Appelo 2006), and the problems subscale of the clinical outcomes in routine evaluation-outcome measure (CORE-OM; Evans et al. 2002). Scores on questionnaires were standardized to a common metric: T-scores with at pretreatment *M* = 50; *SD* = 10 (McCall 1922). In addition, scores were transformed in order to get a normal distribution and a true interval scale, required for calculation of pre-to-posttreatment change scores (de Beurs 2010). In previous studies we compared these instruments on their responsiveness to change and found some variation, amounting to a 10–15% difference in outcome between pairs of instruments (de Beurs et al. 2012).

### Methods for Rendering Treatment Outcome

Treatment outcome was defined by the pre-to-posttreatment difference in severity of symptoms (Delta T or ΔT). Posttreatment scores and ΔT were corrected for case mix differences. The continuous nature of the T-scale optimizes statistical power and simplifies ranking of MHS providers (de Beurs et al. 2016). However, a limitation of ΔT is that it produces a rather abstract figure, which does not yield any information on quality and nature of a patient’s clinical end state. An alternative method to denote treatment outcome was proposed by Jacobson and Truax (1991). Their two core concepts are the reliable change index (JT_RCI_) and clinical significance (JT_CS_). For JT_RCI_ it should be unlikely (*p* < .05) that change as expressed in the difference between the pre- and post-test score is due to measurement imprecision. For the JT_RCI_ a value of ΔΤ = 5.0 is used that represents half a standard deviation and is considered the minimal clinically important difference (de Beurs et al. 2016; Norman et al. 2003; Sloan et al. 2005). Patients with a case mix corrected ΔT > 5 were considered improved. To meet clinical significant outcome or recovered status a patient’s score needs to be changed within the criteria of the reliable change index (JT_RCI_), but also the posttreatment score needs to be within the functional range (JT_CS_). For JT_CS_ a cutoff point of T = 42.5 was determined (de Beurs et al. 2016). Combined with case mix correction, for JT_RCI&CS_ the case mix corrected posttreatment score needs to be T < 42.5 as well as more than five points less than the pretreatment T-score.

### Cost and Treatment Duration

Costs were defined as direct and indirect cost of therapist time for patient care. It involves the costs for diagnosis treatment combinations specified in reimbursement rates in the Dutch fee-for-service system (diagnose-behandeling-combinaties or DBCs as they are called in Dutch (Tan et al. 2012). These data are embedded in 13 treatment time categories of the DBC-code system. Four categories cover short treatment (0–99, 100–199, 200–399, and ≥400 min). Nine categories cover more comprehensive treatment, 250–799, 800–1799, 1800–2999, 3000–5999, 6000–11,999, 12,000–17,999, 18,000–23,999, 24,000–29,999, and ≥30,000 min). These categories were recoded into 13 monetary values based on the cost rates for Dutch MHS in 2014. The cost of treatment for the second year was added to the cost of the first year to arrive at the sum of cost for the complete treatment trajectory for each patient. Cost for psychiatric hospitalizations, for medications, or for treatment by GP’s are not included in this study, as it focusses on outpatient treatments provided by psychiatrists and psychologists who are employed by MHS providers. The cost of a treatment trajectory was on average about 3150 euros (see Table 3).

Treatment duration was calculated in weeks, based on the interval between the date of opening the DBC (usually the first face-to-face diagnostic contact of the patient with the intaker/therapist) and the date of the last face-to-face treatment session. Thus, a possible waiting period between the intake and first treatment session was included in the treatment duration. Both SBG and the Dutch Healthcare Authority (NZa, https://www.nza.nl/organisatie/sitewide/english) provide guidelines and detailed specifications on timing of assessments, on how the start and conclusion date of treatments should be recorded, and on how to log treatment time (the number of minutes). As scrutiny is critical for a fair remuneration system, compliance is monitored through yearly audits by accountants.

To examine the improvement rate over time, duration per outcome was calculated by dividing standardized duration by standardized outcome (both variables transformed to T-scores, to avoid 0-scores in the nominator and denominator). The resulting indicator had value 1 when treatment duration and outcome are in balance. When the indicator value was <1 it took less time to achieve a similar outcome or a better outcome was achieved in the same time. A value above 1 indicated that it took more time or less improvement was achieved. In a similar vein, cost per outcome was calculated by dividing standardized cost by standardized outcome. Thus, the longer and/or more expensive the treatment and/or the lower the ΔT (a worse outcome), the higher these indicator values will be. Both indicators were calculated for each patient (patient-oriented).

In addition, duration and cost per outcome was calculated for each MHS provider by dividing the mean scores on these variables. In addition, duration and cost per reliably improved patient, and duration and cost per recovered patient were calculated by dividing the average cost of the treatment for each MHS provider by the proportion of patients with reliable improvement (JT_RCI_) or with recovery (JT_RCI&CS_). For example, if an MHS provider is able to achieve a 25% recovery rate and their average cost of treatment is 2500 euros, cost per recovered patient is 2500/25% = 10,000 euros; if the recovery rate is 50%, the indicator cost per recovery would be 5000 euros. In a similar way, duration per reliably improved patient was calculated by dividing the average duration by the proportion of patients with reliable improvement (JT_RCI_). If the average duration of treatment for a MHS provider is 30 weeks and the improvement rate is 50%, the indicator “duration per improved patient” is 30/50% = 60 weeks; with 75% improved patients the indicator value is 30/75% = 40. These six indicators are “service provider-oriented”, as they are derived from the average scores on performance indicators achieved by the MHS providers.

### Case Mix Correction

As we expected differences in case mix among MHS providers, various demographic and clinical variables were collected. Socio-economic status (SES) and urbanization was coded in five levels (higher scores indicate higher urbanization or higher SES level) and was derived from the first four digits of the postal codes of patients. Diagnostic information was obtained according to the DSM-IV (American Psychiatric Association 1994), pretreatment disorder severity was operationalized with the pretreatment T-score, and pretreatment functioning with the global assessment of functioning (GAF) scale of the DSM-IV.

There were substantial differences between providers in pretreatment severity of the patients (*F* (7, 3583) = 19.03, *p* < .001, *η* ^*2*^ = 0.04) and GAF-score (*F* (7, 3561) = 91.99, *p* < .001, *η* ^*2*^ = 0.15). Furthermore, the gender distribution differed between providers (χ^2^ (7) = 35.06; *p* < .001), and their populations also differed in age (*F* (7, 3583) = 17.53, *p* < .001, η^2^ = 0.03), socio-economic status (*F* (6, 3337) = 32.12, *p* < .001, *η* ^*2*^ = 0.06), and urbanization (*F* (6, 3344) = 63.99, *p* < .001, *η* ^*2*^ = 0.10). See Table 1 for full details including Bonferroni corrected multiple comparisons of MHS providers on pretreatment severity.

As the populations of MHS providers diverged, all indicators (outcome, duration, and cost) were corrected for case mix differences (Iezzoni 2013). In previous analyses, the pretreatment score appeared the most important case mix variable, explaining about 25% of variance in the posttreatment score (Warmerdam et al. 2016). A higher pretreatment level predicts both a higher posttreatment level as well as a larger ΔT, as it leaves more room for improvement. In addition, outcome was corrected for two other predictors: GAF score and SES. For both items, a lower score was associated with worse outcome. Other variables (e.g. gender or urbanization) were not associated with outcome. This model explained a substantial 29.0% of posttreatment variance in the national dataset (*N* = 29,395) (Warmerdam et al. 2017). Case mix corrected ΔT was calculated by correcting the posttreatment level for case mix variables.

Duration was corrected for initial severity level, functioning, age, gender, and diagnoses. Many of these variables showed different associations with outcome. The diagnoses “major depressive disorder, single episode” and “other mood disorder” were associated with shorter treatment; OCD was associated with longer treatment. A higher severity and worse functioning at pretest were associated with longer treatment. A higher age and male gender predicted longer treatment. This model explained only 2.7% of variance in duration.

Cost was corrected for initial severity level, functioning, age and for the diagnoses “major depressive disorder, recurrent” and OCD. In all these variables, a higher score was associated with higher costs. The model explained a modest 8.4% of variance in cost, which is less than typically found in MHC (Hermann et al. 2007; Iezzoni 2013). This may be explained by diminished variance in the predictors, due to selection of a diagnostically homogenous patient group and by diminished variance in the cost variable, as only outpatient treatments were selected. Finally, potentially relevant variables, such as comorbidity, were assessed with insufficient reliability to be included; others, such as education or living situation had to many missing values (>25%).

### Statistical Analysis

The various performance indicators were compared in patient-oriented and service provider-oriented data. First, treatment outcome of patients was compared among service providers with a repeated measures ANOVA. After this omnibus test, we performed post-hoc tests (all possible pairwise comparisons between providers with a Bonferroni correction for multiple testing) to ascertain which service providers had statistically different outcomes. Mean treatment duration, cost of treatment, duration per outcome and cost per outcome of patients were also compared among service providers with ANOVA. Differences in proportions of recovered and improved patients between service providers were tested with Chi-square tests. The association between duration, cost, and outcome was assessed with correlational analysis (Pearson *r*). Next, service providers were rank ordered according to each performance indicator (service provider-oriented data). To investigate discordance among indicators (or their potential redundancy, because of concordance) we calculated the correlation between rank ordering of the service providers (Spearman *rho* rank correlation coefficient). Finally, we investigated the ability of the indicators to discriminate between service providers with stepwise discriminant analysis. As indicators are correlated (as cost and duration do), two stepwise discriminant analyses were done: one focusing of cost and the other on duration. The option of stepwise entry based on Wilks Lambda was chosen, entering discriminant variables one by one only after they appear to improve the discriminant function significantly. The classification variable is the service provider, indicators are independent variables, and each analysis tests which indicators discriminate best between service providers. The first variable to enter maximizes separation among the groups, the next to enter adds the most in further separating the groups, etc.

## Results

ANOVA of case mix corrected ΔT-scores revealed a significant difference among service providers: *F* (7, 3583) = 38.52, *p* < .001, *η* ^*2*^ = 0.070. Pairwise comparisons showed that providers 1, 2, and 3 differed from all the other service providers (larger ΔT scores), and service provider 8 differed from 4 to 5 (smaller ΔT score). We refer to Table 2 for an overview of the corrected ΔT-scores of the service providers. Outcome diverges considerably between service providers from ΔT = 17.02 for service provider 1 to ΔT = 5.75 for service provider 8, an almost threefold difference in average reduction of symptomatology.

**Table 2:** Treatment outcome (case mix corrected ΔT-score) and percentages of improved and recovered patients for eight providers

Provider	ΔT-score^a^		Improved (JT_RCI_)	Recovered (JT_RCI&CS_)
	M	SD		
1	17.02	13.11	76.9	53.8
2	14.45	14.10	0.1	53.9
3	13.01	11.74	63.0	36.6
4	9.40	11.49	60.3	40.6
5	8.87	11,31	59.2	35.5
6	8.41	13.25	54.5	33.6
7	6.07	10.81	52.2	29.8
8	5.75	12.32	60.3	36.0
Total	11.28	13.31	66.4	43.6
Rank order^b^	1-2-3-4-5-6-7-8		1-2-3-4/8-5-6-7	2-1-4-3-8-5-6-7

$$JT_{{RCI}} = \frac{{M_{{pretest}} - M_{{posttest}} }}{{SEM}};\;SEM = SD \times \sqrt {1 - r_{{ii}} } ; JT_{{CS}} = \frac{{SD_{{pre}} M_{{post}} + SD_{{post}} M_{{pre}} }}{{SD_{{pre}} + SD_{{post}} }};\;{\text{JT}}_{{{\text{RCI}}\&{\text{CS}}}} = {\text{ JT}}_{{RCI}} \wedge {\text{JT}}_{{{\text{CS}}}} ;{\text{ M}}_{{{\text{posttest}}}} ,{\text{ M}}_{{{\text{post}}}} = {\text{ case mix corrected posttest score}}$$

*JT*_*RCI&CS*_ reliable change and clinical significance

^a^Bonferroni corrected pairwise comparisons: 1 to 3 > 4 to 8; 4, 5 > 8

^b^Ranked from lowest to highest ΔT score, from more to less improved and from more to less recovered patients

Table 2 also presents proportions of improved and recovered patients for the service providers and their subsequent ranking. For the categorical outcomes of JT_RCI_ and JT_RCI&CS_, Chi-square tests revealed differences between service providers. For the JT_RCI_ the Chi-square test was *χ* ^*2*^ (7, *Ν* = 3591) = 120.0, *p* < .001 and for the JT_RCI&CS_ *χ* ^*2*^ (7, *Ν* = 3591) = 119.2, *p* < .001. Visual inspection reveals that more patients are reliably changed and recovered by service providers 1 and 2 and less by service providers 6 and 7. Service providers 4 and 8 are a tie. The ranking of service providers according to ΔT and JT-indices generally converges (Spearman *rho* = 0.96; *p* < .01; *N* = 8 between ΔT and JT_RCI_ and *rho* = 0.97; *p* < .01; *N* = 8 between ΔT and JT_RCI&CS_, respectively).

Table 3 presents results regarding duration and costs for the eight providers. For the comparison of the mean treatment duration among service providers, a one-way ANOVA was performed which showed that the service providers differ significantly, *F* (7, 3591) = 41.56, *p* < .001, *η* ^*2*^ = 0.075. Post-hoc tests showed that service provider 1, 2, 5, and 6 treated significantly shorter than service providers 3, 4 and 8. Moreover, 2 treated shorter than 5, 6, and 7; 3 and 4 longer than 5 and 6, 4 longer than 7, and 8 longer than 5 to 7. Average treatment duration ranges from 34.86 to 48.25 weeks, a 1.4-fold difference.

**Table 3:** Mean duration and costs of treatment per provider (case mix corrected, patient oriented data)

Provider	Duration (weeks)^a^		Cost (euros)^a^		Duration/outcome^b^		Cost/outcome^c^	
	M	SD	M	SD	M	SD	M	SD
1	39.55	16.04	2647.14	1520.49	0.93	0.21	0.93	0.19
2	34.86	16.16	1826.70	854.18	0.94	0.29	0.93	0.25
3	47.56	24.74	4315.62	5174.04	1.07	0.32	1.07	0.34
4	47.00	17.87	3508.93	3779.08	1.13	0.29	1.09	0.31
5	40.72	23.54	3686.32	3520.76	1.06	0.29	1.10	0.27
6	39.76	23.27	4409.81	6812.65	1.09	0.45	1.17	0.50
7	42.41	20.86	4376.40	4289.52	1.14	0.34	1.20	0.38
8	48.25	22.53	4195.18	4229.26	1.22	0.37	1.20	0.36
Total	41.06	20.71	3143.51	3777.32	1.05	0.34	1.05	0.33
Rank order^d^	2-1-6-5-7-4-3-8		2-1-4-5-8-3-7-6		1-2-5-3-6-4-7-8		1/2-3-4-5-6-7/8	

^a^Bonferroni corrected pairwise comparisons: For duration: 1, 2, 5, 6 < 3, 4, 8; 2 < 5, 6, 7; 7 < 4, 8. For cost: 1 < 3, 6, 7, 8; 2 < 4 to 8, 3 > 4. For duration per outcome: 1, 2 < 3 to 8, 8 > 3 to 6. For cost per outcome: 1, 2 < 3 to 5; 1 to 4 < 6 to 8; 5 < 7, 8

^b^Standardized duration divided by standardized outcome

^c^Standardized cost divided by standardized outcome

^d^Providers are ranked from low to high duration and from low to high cost

Regarding cost of treatment, a one-way ANOVA showed that the service providers differ significantly, *F* (7, 3591) = 49.09, *p* < .001, *η* ^*2*^ = 0.088. Post-hoc tests indicated that service providers 1 and 2 differ from 6 to 8 (lower costs), service provider 1 differs from 3 (lower costs), service provider 2 differs from 4 to 5 (lower costs) and 3 differs from 4 (higher costs). As cost data were skewed to the right, we also performed a Kruskal–Wallis test revealing again a statistical difference between providers (χ^2^(7) = 565,54, p < .001), with a similar ranking (2-1-6-4-3-5-7-8). Table 3 shows the average costs of service providers, which vary considerably, ranging from *M* = 1827 to 4410 euros. This revealed a 2.4-fold difference between service providers with the lowest and the highest average cost per treatment.

Table 3 also presents results of the indices combining duration or cost with outcome. Service providers differ significantly on the duration per outcome indicator: *F* (7, 3591) = 50.61, *p* < .001, *η* ^*2*^ = 0.090. Post-hoc tests indicated that service provider 1 and 2 scored better on this index than all other service providers. Service provider 7 and 8 differed from all other service providers showing longer treatment duration (see Table 3). Service providers also differ significantly on the cost per outcome index: *F* (7, 3591) = 59.67, *p* < .01, *η* ^*2*^ = 0.104. Post-hoc tests indicated that again service provider 1 and 2 had lower cost per outcome compared to most of the other service providers. Eight had higher costs per outcome than the service providers 1 to 5.

Next, for each service provider mean duration divided by mean outcome, duration and cost per improved and duration and cost per recovered patient were calculated. Table 4 presents an overview of how service providers ranked on these indices. The results reveal a difference between service providers on the index duration per outcome, ranging from 2.32 to 8.39 weeks per ΔT unit (≈0.1 *SD*). Thus, these service providers diverge by a factor 3.6. Put differently: it takes on average 3.6 times longer to achieve one ΔT-point improvement for service provider 8 compared to service provider 1. Variation in treatment duration per improved patient among service providers is also substantial, with the fastest and the slowest service provider differing by a factor 1.8 (45.81 vs. 81.25 weeks for service provider 2 and 8, respectively). In duration per recovered patient these service providers diverge by a factor 2.2 (64.68 vs. 142.32 weeks).

**Table 4:** Overview of case mix corrected duration and costs per outcome indicator (provider-oriented data)

Provider	Duration per			Cost per		
	ΔT-unit	Improved patient	Recovered patient	ΔT-unit	Improved patient	Recovered patient
1	2.32	51.43	73.51	155.49	3 442	4 920
2	2.41	45.81	64.68	126.45	2 400	3 389
3	3.66	75.49	129.95	331.72	6 850	11 791
4	5.00	77.94	115.76	373.38	5 819	8 643
5	4.59	68.78	114.70	415.56	6 227	10 384
6	4.73	72.95	118.33	524.56	8 091	13 124
7	6.99	81.25	142.32	721.14	8 384	14 686
8	8.39	80.02	134.03	729.23	6 957	11 653
Total	3.64	61.84	94.17	278.68	4 734	7 210
Rank order^a^	1-2-3-5-6-4-7-8	2-1-7-6-4-3-5-8	2-1-7-3-6-4-5-8	2-1-3-4-5-6-7-8	2-1-3-7-4-5-6-8	2-1-3-7-5-4-6-8
Factor^b^	3.5	1.8	2.2	5.8	3.5	4.3

^a^Providers are ranked from low to high duration and from low to high cost

^b^Highest value divided by the lowest value

Cost per outcome ranges between service providers from € 126.45 for service provider 2 to € 729.23 per ΔT unit for service provider 8, a 5.8-fold difference between the most and the least cost-efficient service provider (see Table 4). In terms of cost per reliably changed or recovered patient, service providers differed as well. Here, service provider 2 diverges the most from service provider 7 with for change or recovery, a factor 3.5 and 4.3, respectively. Thus, to achieve recovery is on average almost 4.3 times costlier with service provider 7 (14 686 euros) than with service provider 2 (3 389 euros).

Next, we investigated associations among indicators. Pearson correlation of patient-oriented data between duration and costs showed that duration of treatment was substantially related to the cost of treatment (*r* = .42; *p* < .001; *N* = 3591). There was no association between outcome and duration of treatment (*r* = −.03; *p* = .05; *N* = 3591). We detected a minor negative association between outcome and cost (*r* = −.05; *p* < .01; *N* = 3591). The costlier treatments yield slightly lower ΔT scores. Table 5 presents Spearman rho coefficients of service provider-oriented data for the concordance between performance indicators in rank order of service providers. The ranking of service providers according to ΔT generally converges with the ranking according to cost-indicators, but less so with duration. Some associations are not statistically significant, due to the small *N* = 8 for service providers. There is a high concordance between ranking based on ΔT and cost per outcome.

**Table 5:** Association of ranking according to DT with ranking according to other performance indicators (*N* = 8)

Duration	0.57^ns^	Cost	0.69^ns^
Duration per DT	0.88**	Cost per DT	0.98**
Duration per improved	0.57^ns^	Cost per improved	0.83*
Duration per recovered	0.67^ns^	Cost per recovered	0.81*

*ns* not significant

*p < .05; **p < .01 (2-tailed)

Finally, we compared the ability of indices to discriminate between service providers with stepwise discriminant analysis. The first discriminant analysis evaluated three indicators: outcome, cost, and cost per outcome. A stepwise discriminant analyses revealed that the cost per outcome indicator entered the discriminant function first (canonical correlation *r* = .371), followed by cost (*r* = .142), and outcome (*r* = .063). Apparently, and in line with the other findings, cost per outcome maximizes separation among the service providers. The discriminant function could classify 41.9% of the cases to the correct service provider. The second discriminant analysis evaluated outcome, duration and duration per outcome. Again, the index combining information on duration and outcome entered first (canonical correlation *r* = .349), followed by duration (*r* = .161) and outcome (r = .076). This discriminant function classified 41.8% of the cases to the correct service provider.

## Discussion

The prime aim of the present study was to compare performance indicators on how they distinguish between service providers. Specifically, we investigated performance indicators that combine outcome with treatment process variables, such as duration and costs of treatment, as compared to looking at outcome alone. When comparing the performance indicators, it seems at first glance that information on cost does not matter much for ranking, considering the high association between ΔT and cost per ΔT (Table 5). Including cost in the performance indicator does however, amplify the differences among service providers. They diverge more on duration per ΔT (a 3.5-fold difference between the highest and lowest indicator value) or cost per ΔT (a 5.8-fold difference) than on ΔT alone (a threefold difference). In other words, differences between service providers in performance are larger when also duration and/or cost are considered. With categorical outcomes, combining costs and outcomes augments differences among service providers as well. The service provider with the best results is 4.3 times more efficient than the provider with the least favorable results, when we look at the cost-for-recovery indicator, which is on average € 3389 against € 14,686 per recovered patient. The results regarding the association among performance indicators reveal that outcome and cost per outcome lead to more similar rankings than outcome and duration per outcome, especially when we consider cost per improved or cost per recovered patient. Performance indices do not overlap completely; rather, they add information onto each other. Finally, discriminant analyses revealed that the combined indices of cost per outcome and duration per outcome maximized separation among service providers. Apparently, efficiency discriminates better between service providers than outcome only and yields a more informative and useful performance indicator.

The cost per outcome indicator allows for a straightforward ranking of the service providers regarding their efficiency. However, cost per outcome is by itself a rather abstract concept and not very appealing from a clinical perspective. Looking at the cost of improvement or recovery is less abstract and a more informative way to look at efficiency. Yet, a potential drawback of looking at outcome categorically is that statistical power to find differences among groups diminishes (Fedorov et al. 2009), which could prove problematic when smaller datasets are used. We suggest that these indices be used to illustrate findings, by examining first the general picture provided by the cost per outcome based on the continuous scale (ΔT) and then moving on to the more appealing cost per reliable improvement or cost per recovery.

At the service provider level duration and cost are negatively associated with outcome. Service providers who provide shorter and less expensive treatments also tend to have better outcome in terms of pre-to-posttreatment change in severity of symptoms. For example, service provider 8 is procuring the highest cost and is also the one with the lowest improvement stats. Consequently, service provider 8 is consistently last in the rankings based on the ratio of cost per outcome across the indices. On the other hand, service provider 2 has the least costs while showing the highest number of improved patients and is consistently first in the ranking based on the ratio of cost per outcome across indices. By and large, the service providers with the highest change scores (the best results) also treat shorter and procure lower costs, despite case-mix correction of these indicators.

At the patient level, irrespective of service provider, the association between outcome and duration or cost is no longer found. A daring explanation for this disparity in findings is that the association between duration or cost with outcome is not contingent upon patient characteristics, but rather on service provider characteristics. Quite consistently, the service providers who rank first and second have better results than the other service providers, both in posttreatment outcome and in the proportion of recovered or merely changed patients. In contrast, the service provider who ranked last had the lowest scores on all three outcome indices. This service provider had a smaller ΔT score and less patients in the recovered or changed categories.

It is unknown which factors cause variation in efficiency and outcome. Service providers differ in the kind of treatments that are offered (e.g., in accordance with evidence based guidelines vs. experience based), how well treatments are provided by professionals and assignments executed by patients (treatment integrity), flexibility in treatment intensity, how frequently ROM is applied and which measure is used, and in institutional culture, or “corporate” management (Anderson 2006), to mention a few. For instance, some service providers do not offer inpatient treatment whereas others do. Service providers who do not provide inpatient treatment may refer more severe patients out which may boost their results. Some service providers, apply ROM only at pre- and posttreatment, whereas others monitor more frequently, allowing for timely adjustment of treatments that are “not on track” and likely result in treatment failure (Lambert 2010). Organizational differences between service providers will also influence treatment duration, cost, and outcome. For example, some service providers explicitly aim for short protocolled cognitive-behavior therapy treatments (usually concluded within a year) or put extensive effort in timely ending of unproductive treatments through so-called “outtake” teams (Hoogduin et al. 1997; Verbraak and Hoogduin 2013). Termination of treatment can be difficult, especially when insufficient improvement has been achieved, as it runs counter to the professional ethos of caring therapists (Cohen et al. 2006). Yet, continuing an unproductive treatment is not beneficial to the patient either and diminishes the efficiency of care.

Comparing outcomes between service providers calls for caution and prudence given the observational nature of the present data. Firstly, service providers used different outcome measures and had different rates for the completeness of pre-to-post assessments. Furthermore, nonrandom missingness of outcome data (e.g., because patients who prematurely terminate treatment are less inclined to comply with posttest assessments) may bias the results of providers, reduce information validity, and hamper comparison (Cuddeback et al. 2004). Also, service providers differ in the treatment modalities they offer, with some providing at least some inpatient service, inducing substantial divergence in cost, and potentially influencing the type of patients referred to them. In addition, service providers vary substantially regarding the patient population they serve, their so-called case mix. Some patients are harder to treat than others; they require more intensive treatment with longer and/or higher costs, but may still have worse outcomes. Although we did correct all indicators of outcome, duration and cost for known case mix differences among service providers (Iezzoni 2013; Warmerdam et al. 2016), one could still argue that case mix correction is a statistical band-aid, never perfect, and potentially deficient. Relevant case mix variables may have been overlooked or are still unknown. For instance, prior episodes of psychopathology or prior treatments may be an impediment to good outcome, but this information was not available. The only way to fully rule out the influence of known and unknown pretreatment differences between compared populations is a true experimental design with randomization (Hulley et al. 2013). As no randomization of patients to service providers has taken place in this observational study, results should be interpreted with outmost caution regarding comparative performance of service providers. Only a truly experimental study design will yield conclusive answers to the question of how cost or duration of treatment and outcome are interrelated and whether duration of treatment is dependent on patient or service provider characteristics.

The main strength of this study is the use of real life data, not acquired in a clinical trial with selected patients, but under the daily circumstances of clinical care provision. This allows to reach conclusions that are based on real-life clinical practice and increases our confidence in the generalizability of the results regarding the usefulness of indicators. A further strength of this study is that it is based on a large dataset. The dataset consists of the treatment trajectories of over 3000 patients from eight mental health service providers. It constitutes a representative mix of integrated service providers and providers who specialize in only outpatient treatment. Patients from various age, gender, diagnostic, educational and socio-economic groups were included representing the outpatient population well. In addition, from a statistical point of view, the sample size ensures adequate statistical power for the required analyses. We have limited the present analysis to treatments with a maximum duration of 2 years, thereby excluding about 10% of the treatments, which took longer than 2 years to complete. Omitting them has likely affected the results. The excluded long treatments occurred predominantly in service providers with on average longer treatments. Therefore, the present results may be a too conservative representation of the true differences between service providers, as outliers were mostly excluded in service providers with poorer results.

A limitation of the study is that comparison of service providers is hampered, as their outcomes may be confounded by some patient characteristics we did not correct for, such as previous psychopathology and treatment history. Furthermore, the study is limited to short term outcome, assessed immediately after the conclusion of treatment. One may argue that lengthier, more expensive treatments may yield more lasting results in the long run, whereas shorter treatments might have a higher relapse/recurrence rate (Bockting et al. 2005; Vittengl et al. 2007). Future studies should broaden the scope by following how patients fare in the period after conclusion of treatment. This requires the collection of follow-up data, for instance 6 months or 1 year later regarding psychological health and use of (mental) health care. It would be interesting to investigate how cost-effectiveness and relapse rates are associated, as more extensive (and expensive) treatments may be offset by better results in the long run.

We have included waiting time prior to the treatment in the treatment duration. Service providers vary considerably in waiting time. The average per service provider ranges from 2.1 to 8.4 weeks. One could argue against our choice to include waiting time, since being on a waitlist is not the same as undergoing treatment. Yet, from the perspective of the patient a long waiting time is adverse and does prolong the time till improvement or recovery. Thus, for a fair comparison between service providers, it is in our opinion correct to have included waiting time in the duration of treatment variable.

It should be stressed that the purpose of the present study was not a formal cost-effectiveness or cost benefit analysis, but rather a comparison of patient-oriented performance indicators. The study approached efficiency from a patient (duration) and service providers’ (cost) perspective and the results can only be interpreted in that context. Different conclusions may be drawn when effectiveness and efficiency are approached from the broader societal perspective, which would imply the gathering of extra data regarding cost of illness, financial benefits of treatment and gains in quality adjusted life years (van Agthoven et al. 2014).

For future studies, it would be useful to examine the applicability of the effectiveness and efficiency indicators on inpatient treatment, as the present study concerned predominantly outpatient treatments. Although inpatients with severe mental disorders form a relatively small subgroup in MHS in The Netherlands (about 15%), they put a disproportionate burden on the finances, as inpatient treatment is up to ten times more expensive. Furthermore, the source of differences in efficiency among treatment providers should be investigated further, to examine whether these differences are associated to demographic features, institutional culture or factors related to the treatment process, as the latter two are key to quality improvement and reducing clinical variance. There was quite some variation in treatment duration. Future studies could examine the association between duration of treatment and outcome in more detail. For example, by investigating whether patients who require lengthier treatments differ in baseline characteristics from those who require less treatment or whether the lengthiest treatments are predominantly treatment failures.

In times of rising health care costs and finite budgets for health care, the quest for more efficient MHS delivery is opportune and swift positive results are beneficial to our patients. In The Netherlands, most service providers offer a standard treatment regime of weekly or biweekly sessions and are—for logistic reasons—not inclined to attune the session frequency to the momentary needs of patients. More flexibility, for instance by offering more intensive treatment in the initial phase or a more varied treatment menu (group therapy, e-health applications and blended forms) may yield speedier, better, and more lasting results. We may improve MHS with quality management, by testing and evaluating such organizational interventions in Plan-Do-Study-Act (Deming 2000) cycles.
